# Drawing and Soccer Tactical Memorization: An Eye-Tracking Investigation of the Moderating Role of Visuospatial Abilities and Expertise

**DOI:** 10.3390/vision10010002

**Published:** 2026-01-01

**Authors:** Sabrine Tlili, Hatem Ben Mahfoudh, Bachir Zoudji

**Affiliations:** Laboratoire de Recherche Sociétés & Humanités, DeVisu Department, Polytechnic University of Hauts-de-France, 59300 Valencienness, France; sabrine.tlili@uphf.fr (S.T.); bachir.zoudji@uphf.fr (B.Z.)

**Keywords:** drawing, visuospatial abilities, expertise, memorization, eye-tracking

## Abstract

Dynamic drawing has emerged as a strategy to communicate tactical diagrams, yet its effectiveness remains uncertain and appears to depend on individual differences. This study investigated how the memorization and visual processing of tactical soccer scenes are influenced by drawing format (static drawing vs. dynamic drawing) and moderated by visuospatial abilities (VSA) and expertise. Expert (N = 57) and novice (N = 54) participants were randomly assigned to one of two conditions. In the static drawing condition, participants viewed a pre-drawn, completed tactical diagram accompanied by an oral explanation. In the dynamic drawing condition, they observed the coach drawing the diagram in real time while delivering the same explanation. VSA was first assessed using a control test. Then, in the main test, participants memorized and reproduced the tactical scene while their eye movements were recorded using an eye tracker. Key findings revealed a three-way interaction, highlighting the occurrence of an expertise reversal effect: high VSA novices performed better with dynamic drawing, whereas low VSA experts benefited more from static drawing, showing distinct visual processing patterns across groups. Overall, the results highlight the need to tailor drawing strategies to individual characteristics, particularly VSA and expertise, to optimize visual attention and tactical memorization.

## 1. Introduction

Learning is mainly shaped by the instructional methods employed rather than by the medium through which content is delivered [1]. To promote optimal tactical learning, it is therefore essential to determine the most effective methods for integrating visual aids. In team sports, coaches often use play diagrams, typically drawn on tactical boards, accompanied by verbal commentary (narration) to convey the key spatial-temporal aspects of tactical instructions [2,3]. According to the cognitive theory of multimedia learning [4,5,6], narrated diagrams are grounded in three principles of human information processing. First, the dual-channel principle states that learners process verbal and pictorial information through separate channels [7]. Second, the limited-capacity principle emphasizes that each channel can handle only a limited amount of information at a time [8,9]. Third, the active-processing principle holds that meaningful learning occurs when learners engage in appropriate cognitive processing by selecting relevant information, organizing it into coherent structures, and integrating it with prior knowledge from long-term memory [10]. Accordingly, memorizing narrated diagrams remains cognitively demanding, especially for novices, due to the need to align auditory information with visual elements, which can increase cognitive load [5,11,12]. In this case, learners often engage in unnecessary visual search to locate relevant information, consuming cognitive resources and hindering learning [12]. In response to these challenges, dynamic drawing has emerged as a promising instructional method [13,14]. The dynamic drawing principle posits that observing the hand-drawn, dynamic creation of static visuals, rather than presenting static, pre-drawn diagrams, enhances learners’ cognitive engagement and improves understanding [13,15,16,17].

### 1.1. Dynamic Drawing Versus Static Drawing

In educational contexts, dynamic drawings accompanied by verbal explanations are recognized as an effective instructional method. They have been shown to enhance learner engagement and comprehension more effectively than static slides, audio, or text alone [18,19]. In addition, Fiorella and Mayer [13] found that learners who viewed real-time dynamic drawings in video lessons achieved better transfer performance than those who viewed static pre-drawn diagrams. Recently, Zhang et al. [20] showed that students who watched hand-drawn dynamic visuals outperformed those who viewed static computer-generated slides in both immediate and delayed post-tests. These findings support the dynamic drawing principle [17] and suggest that this drawing method reduces the extraneous processing of irrelevant information, thereby preserving working memory resources. Consequently, learners can devote more capacity to essential processing, constructing mental representations of key information, and to generative processing by reorganizing the material and integrating it with prior knowledge from long-term memory [21]. Indeed, these benefits stem from the alignment of dynamic drawing with several multimedia learning principles. Particularly, consistent with the signaling (or cueing) principle, which posits that learning improves when key information is highlighted [22,23], the instructor’s hand acts as a visual cue guiding attention to relevant elements of the diagram. Moreover, the segmenting principle, which emphasizes that dividing a multimedia lesson into manageable units facilitates learning [24], is reflected in the gradual drawing of the diagram’s parts. Furthermore, the temporal contiguity principle, which holds that learning is enhanced when spoken explanations and visuals are presented simultaneously [22,25], is upheld by synchronizing the instructor’s speech with the drawing process. On the other hand, in a pioneering study on drawing and tactical soccer learning, Tlili et al. [14] found no significant difference in learning efficiency between dynamic- and static-drawing conditions, as participants performed similarly across conditions. However, notably, despite the absence of a direct drawing effect, the study revealed a moderated effect of visuospatial abilities (VSA), suggesting that the effectiveness of instructional drawing strategies strongly depends on individual cognitive characteristics.

### 1.2. Visuospatial Abilities

VSA, a cognitive trait that varies across individuals, influences learning outcomes, particularly when visual aids are involved [26]. They refer to the capacity to recall, generate, depict, and manipulate symbolic information, involving the mental manipulation of static visuals (static VSA) and reasoning about moving elements (dynamic VSA) [27,28]. Two opposing hypotheses aim to explain the interaction between VSA and visual presentation formats. The ability-as-enhancer hypothesis suggests that high VSA individuals benefit more from dynamic visualizations and outperform low VSA individuals, as their superior cognitive and attentional capacities enable a more efficient processing of complex visual information [29,30,31]. In contrast, the ability-as-compensator hypothesis posits that dynamic visualizations support low VSA individuals by providing external representations that reduce the need for complex mental manipulation, leading to learning efficiency comparable to that of high VSA learners [32,33,34]. The importance of VSA in tactical learning has recently been established in team sports. Ben Mahfoudh and Zoudji [35] reported that high VSA players demonstrated greater learning efficiency in memorizing dynamic tactical scenes, needing fewer repetitions and expending less mental effort than low VSA players. In addition to learning efficiency, VSA also play a key role in shaping the visual processing of tactical soccer scenes [36]. This visual attentional process relies on two fundamental gaze metrics: fixations, which are periods of relative ocular stability allowing visual information to be extracted, and saccades, which are rapid ballistic eye movements that shift the gaze from one fixation point to another. Eye-tracking studies revealed that high VSA players exhibited longer fixations and slower, shorter saccades, reflecting focal processing and sustained attention. In contrast, low VSA players exhibited shorter fixations and faster, longer saccades, indicating ambient processing that impeded the conscious identification of key elements [36,37,38,39]. Within the realm of drawing methods, Tlili et al. [14] emphasized the moderating role of VSA in learning soccer tactics. Their findings showed that as VSA increased, the benefits of dynamic drawing on tactical scene memorization became more evident, supporting the “ability-as-enhancer” hypothesis. However, the main limitation of that study was the lack of consideration for learners’ expertise, despite evidence that instructional effectiveness depends on prior knowledge.

### 1.3. Expertise and Prior Knowledge

Expertise level is a key factor influencing athletes’ performance in multimedia learning contexts. Indeed, their domain-specific prior knowledge, built through sustained and deliberate practice [40,41], enables experts to be more efficient than novices at memorizing new information and managing cognitive load [42,43]. Experts adeptly encode and recall game patterns by organizing information into meaningful chunks stored in long-term memory (LTM) and treating it as a single unit [44,45]. In tactical learning, previous studies have reported higher learning efficiency among experts compared to novices, as experts remembered the scene better with fewer repetitions and less mental effort [46,47]. Additionally, experts are characterized by selective attention [48], which enhances the visual processing and learning of tactical information. Eye-tracking evidence shows that, compared to novices, experts demonstrate longer fixations as well as longer and faster saccades, allowing them to quickly locate and process relevant information [37]. This pattern reflects a top-down mechanism whereby learners with higher prior knowledge focus on relevant information and ignore irrelevant details [3]. In contrast, novices rely on bottom-up processing and require external guidance to focus their attention efficiently [49]. Although both top-down and bottom-up guidance reduce visual search difficulties, their overlap may induce the expertise reversal effect as external guidance can impose unnecessary processing demands on experts, making methods effective for novices less beneficial or even counterproductive [47,50]. In the framework of drawing methods, the positive impact of dynamic drawings on learning in academic settings has been observed mainly among novices, suggesting a boundary condition linked to prior knowledge, as students with with high prior knowledge do not benefit from dynamic drawings [13]. However, the effect of drawings on learning has not been examined in relation to the combined influence of expertise and VSA, although their interaction has been explored in tactical learning contexts. For novices, high VSA compensate for limited expertise, allowing performance comparable to experts with low VSA, whereas among experts high VSA play a more nuanced role by reducing the performance gap between experimental conditions [27,51].

### 1.4. Rational of the Study

Although recent research has examined the role of drawing in tactical learning [14], it has yet to consider factors that may moderate its effectiveness. This paper aims to investigate two learner characteristics, VSA and expertise level, that may influence the effectiveness of drawing-based instruction. Based on the aforementioned studies, we first hypothesized that there would be no significant direct effect of the drawing condition on learning efficiency and we also considered that the absence of a dynamic drawing condition effect could be related to the visual processing demands imposed by instructional materials [14]. Secondly, based on research on expertise in tactical learning [46,47] and consistent with the ability-as-enhancer hypothesis [30,31], we expected that experts and high VSA participants would accordingly outperform novices and low VSA participants in memorizing tactical scenes. Experts were expected to benefit from selective attention to relevant information [37,46], whereas high VSA individuals were expected to display focal visual processing patterns [36,37]. Thirdly, based on the expertise reversal effect and the declining impact of VSA with increasing expertise, we predicted that the dynamic drawing condition would benefit novices, particularly those with high VSA, while the static drawing condition would be more suitable for experts, especially those with low VSA. This interaction was expected to be reflected in more efficient visual processing patterns across groups.

## 2. Materials and Methods

### 2.1. Participants

A priori power analysis was conducted using G*Power (Version 3.1.9.7; [52]), based on a medium effect size (f^2^ = 0.15), a power level of 0.95, and an α level of 0.05. Results indicated that a minimum of 89 participants was sufficient to ensure robust statistical validity. To account for potential dropouts or technical issues, 111 male participants were recruited and divided into two groups: 54 novices and 57 experts. The novice group consisted of university students (M age = 21.5 years, SD = 3.3) with no club-level experience in soccer or other team sports. Their limited exposure, restricted to physical education classes or informal games, ensured the absence of cross-sport transfer effects [53]. The expert group (M age = 25.2 years, SD = 2.27) comprised soccer players actively competing at a top level. They had been playing soccer for an average of 12.5 years (SD = 2.43) and trained for an average of 8.5 h per week (SD = 1.8). According to the criteria defined by Swann et al. [54], they were classified as competitive elite athletes. All participants reported no prior experience with similar laboratory experiments and no uncorrected vision problems. Participation was voluntary, with informed consent obtained from all individuals. The study received ethical approval from the Ethics Committee of the affiliated laboratory and the University.

### 2.2. Materials

Two computerized tests were presented on a 15.6-inch laptop. The first was a control test comprising two tasks to assess participants’ VSA. The second was the main test, in which participants memorized a soccer scene and reproduced it on paper. The video-based tactical scene was recorded in two versions using an Ultra HD camera, positioned 1.5 m from the whiteboard. During visualization, eye movements were tracked with Tobii Pro Glasses 2 (50 Hz; Tobii AB, Danderyd, Sweden), a wireless head-mounted eye tracker connected to a 13-inch Dell laptop. Gaze data acquisition and subsequent analysis were carried out using Tobii Pro Glasses Controller (version 1.95) and Tobii Pro Lab (version 1.241), respectively. Before the manual verification of gaze mapping, gaze samples were processed using the Tobii I-VT filter. This filter is among the most commonly used in eye-tracking research [55,56,57] and relies on several algorithms and functions designed to classify fixations and saccades. Each component has a specific role and requires predefined parameter settings. The “I-VT classifier” evaluates the angular velocity associated with each sample and categorizes it as belonging either to a fixation or a saccade. When the sample’s velocity is below the Velocity threshold of 30°/s [57,58], it is classified as part of a fixation; when it reaches or exceeds this value, it is classified as part of a saccade. The “velocity calculator” computes angular velocity between consecutive gaze samples. Its parameter Window length, fixed at 20 ms, defines the temporal interval used for estimating velocity and provides an optimal balance between reducing high-frequency noise and avoiding excessive smoothing of rapid transitions [57]. The “median noise-reduction” function acts as a low-pass filter that smooths noise while preserving key signal features required for accurate classification [59]. It removes noise caused by small head movements, sensor jitter, or transient tracking instability, thereby preventing noise from being misclassified as saccades, while avoiding excessive attenuation of the true saccadic velocity peaks. The “Gap fill-in interpolation” function replaces short gaps in the data caused by tracking loss (e.g., blinks or momentary occlusions). Its parameter Max gap length, set to 75 ms [60,61,62], specifies the maximum duration of a gap that can be interpolated. The “Merge adjacent fixations” function stitches together fixation fragments that have been artificially split. Two fixations are merged when the time separating them is shorter than the Max time between fixations set to 75 ms and when the visual angle between their positions is smaller than the Max angle between fixations set to 0.5° [60,63,64]. Finally, the “Discard short fixations” function removes very brief fixations that are considered non-meaningful. Fixations shorter than the Minimum fixation duration of 60 ms [60,63,64], are thus excluded from the dataset.

### 2.3. Measures

#### 2.3.1. Control Test

Following the taxonomy proposed by Ben Mahfoudh et al. [65], two tests were used to assess static and dynamic VSA. Static VSA was measured using the Mental Rotation Task [66], where participants mentally rotated 2D and 3D figures to identify two correct matches among four alternatives. Dynamic VSA was assessed using the “Shoot” task [67,68], which involved predicting the trajectory of a moving object. Participants launched a black ball vertically (1600 px/s) by pressing the G key, aiming to collide with a white ball moving horizontally (900 or 1400 px/s). A composite VSA score was then calculated by averaging the results of both tasks [51].

#### 2.3.2. Main Test

**Figure 1:**
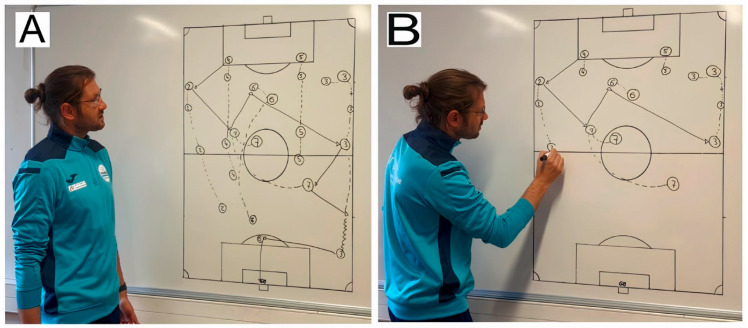
Illustrative images of the two experimental conditions: (**A**) static drawing and (**B**) dynamic drawing.

In the main test, participants were instructed to memorize one of two video-based tactical lessons illustrating an offensive soccer play: either a dynamic drawing or a static drawing version (Figure 1).

**Figure 2:**
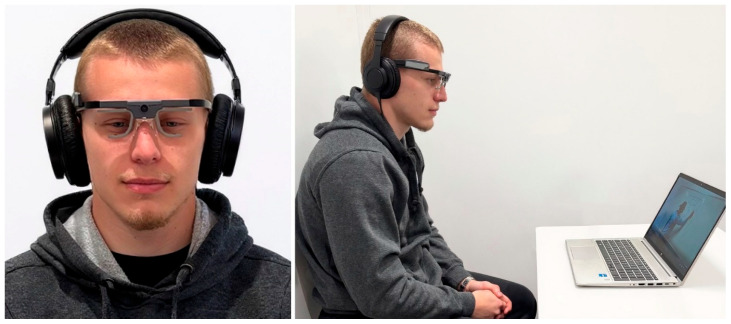
Experimental setup with Tobii Pro Glasses 2 during tactical scene visualization.

In both conditions, a male coach verbally described a six-pass sequence involving six attacking players, ending with a goal. The content and narration were identical across versions, each lasting approximately 3 min. In the static drawing condition, the coach stood beside a pre-drawn diagram and provided commentary. In the dynamic drawing condition, he progressively drew the scene on the whiteboard while explaining it. In both cases, no pointing gestures or gaze guidance were provided. While viewing the video, participants wore Tobii Pro Glasses 2 to record eye movements via infrared-based corneal reflection (Figure 2).

Three gaze metrics were collected: first, average duration of fixations (ADF), indicating how long participants focused on diagram elements, in milliseconds; second, average amplitude of saccades (AAS), measuring the angular distance between fixations, in degrees; and third, average peak velocity of saccades (APVS), reflecting the maximum angular speed reached during each saccadic movement, in degrees per second.

### 2.4. Procedure

Participants were randomly assigned to either the dynamic drawing or static drawing group. They first completed the control test, followed by the main test. Before the visualization phase, a standard eye-tracker calibration was performed. Participants were then instructed to keep their head stable and maintain their gaze on the screen to minimize the risk of excluding a profile, due to long gaps not handled by the built-in gap-fill al-gorithm, and replacing it with another from the same group. After viewing the video, they rated their perceived cognitive load using three 9-point Likert scale items: intrinsic load ‘How much mental effort did you invest?’ [69], extraneous load ‘How difficult was it to learn with the material?’ [70], and germane processing ‘How much did you concentrate during learning?’ [71]. A global mental effort score was computed by averaging the responses. Next, participants completed the recall test by reconstructing the six-action sequence on a printed blank soccer field, placing players and/or the ball in the correct positions. No time limit was imposed, participants worked at their own pace, and the time taken to complete the task was recorded in seconds. An independent evaluator scored the reconstructions, assigning one point for each correct action and each correct placement, with respect to the key reference lines and areas of the field, and zero otherwise [2,14].

### 2.5. Data Analysis

To assess memorization efficiency, three dependent variables, recall accuracy, overall mental effort, and recall time, were used to calculate a composite learning efficiency score based on Tuovinen and Paas’s [72] three-dimensional formula. This approach involves standardizing the raw scores (z-transformation) for each variable.

Learning efficiency = (Zrecall accuracy − Zoverall mental effort − Ztime)/√3

To investigate the direct effect of focal predictors and the moderating effects of VSA and expertise level on the relationship between experimental conditions (dynamic drawing and static drawing) and the four dependent variables, learning efficiency, ADF, AAS, and APVS, we employed Model 3 of the PROCESS macro for SPSS version 25 [73]. This statistical tool, developed by Preacher and Hayes [74], allows for the examination of moderation and conditional effects using ordinary least squares regression [75]. The VSA, as a continuous variable, was mean-centered, and the analyses were performed with 5000 bootstrap samples and 95% confidence intervals, with significance thresholds set at *p* ≤ 0.05. Four separate moderation analyses were conducted, with both VSA and expertise level included as moderating variables and learning efficiency ADF, AAS, and APVS as the dependent variables.

## 3. Results

The descriptive statistics for the four dependent variables across drawing conditions, expertise groups, and VSA levels are reported in Table 1.

### 3.1. Learning Efficiency

**Figure 3:**
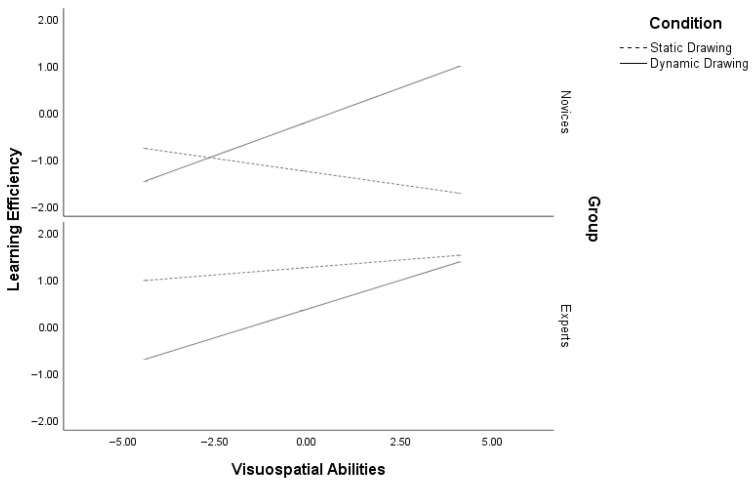
Moderation of learning efficiency by visuospatial abilities and expertise in dynamic and static conditions. Learning efficiency = (Zrecall accuracy − Zoverall mental effort − Ztime)/√3 [72].

The analysis of learning efficiency revealed a significant regression model, R^2^ = 0.61, *p* < 0.001. The regression analysis showed no main effect of condition [*β* = 0.06, se(HC4) = 0.20, *p* = 0.73] but a significant main effect of VSA [*β* = 0.11, se(HC4) = 0.02, *p* < 0.001] and expertise [*β* = 1.59, se(HC4) = 0.20, *p* < 0.001] on learning efficiency. Individuals with high VSA were more efficient than those with low VSA, and experts similarly outperformed novices. Additionally, a significant three-way interaction between VSA, expertise, and condition emerged [*β* = −0.21, se(HC4) = 0.10, *p* = 0.04]. For novices, participants with low VSA learned equally from the static- and dynamic-drawing conditions (*p* = 0.12), whereas those with high VSA (*p* < 0.001) learned better from the dynamic drawing condition. This finding suggests that novices with low VSA do not benefit from either drawing condition, whilethe positive effect of dynamic drawings becomes stronger at relatively higher levels of VSA. For experts, those with low VSA (*p* < 0.001) benefited more from the static- than the dynamic-drawing condition, whereas high VSA experts performed similarly across both conditions (*p* = 0.71). These results indicate that, despite their prior knowledge, low VSA experts do not benefit from dynamic drawing, whilehigh VSA experts are able to process dynamic drawing efficiently and take advantage of both conditions (Figure 3).

### 3.2. Average Duration of Fixations

**Figure 4:**
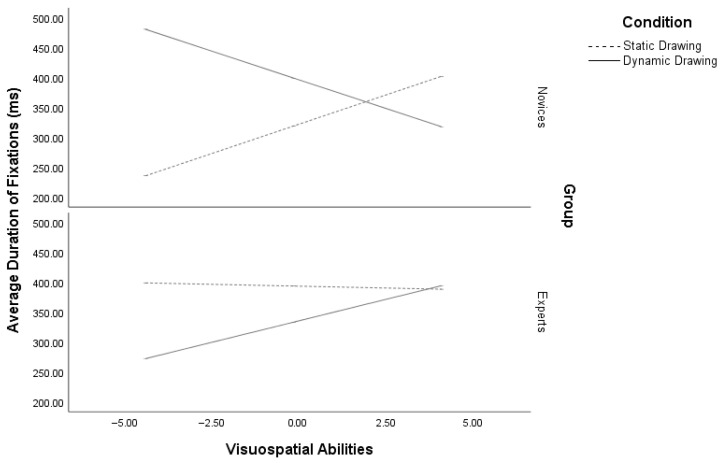
Moderation of average duration of fixations by visuospatial abilities and expertise in dynamic and static conditions.

The analysis of ADF showed a significant regression model, R^2^ = 0.53, *p* < 0.001. The regression analysis revealed a main effect of condition [*β* = 204.47, se(HC4) = 51.71, *p* < 0.001], VSA [*β* = 133.03, se(HC4) = 17.20, *p* < 0.001] and expertise [*β* = 202.24, se(HC4) = 41.02, *p* < 0.001] on ADF. Participants in the dynamic drawing condition, as well as those with high VSA and experts, exhibited longer fixations compared to those in the static drawing condition with low VSA and novices, respectively. Moreover, a significant three-way interaction among VSA, expertise, and condition was observed. [*β* = 54.15, se(HC4) = 7.08, *p* < 0.001]. Low VSA novices (*p* < 0.001) exhibited longer fixations in the dynamic drawing condition compared to the static condition, whereas high VSA novices (*p* = 0.01) showed the opposite pattern, spending more time fixating in the static drawings. Experts with low VSA (*p* < 0.001) demonstrated longer fixations in the static drawings, while experts with high VSA fixated equally on both drawing conditions (*p* = 0.75) (Figure 4).

### 3.3. Average Amplitude of Saccades

**Figure 5:**
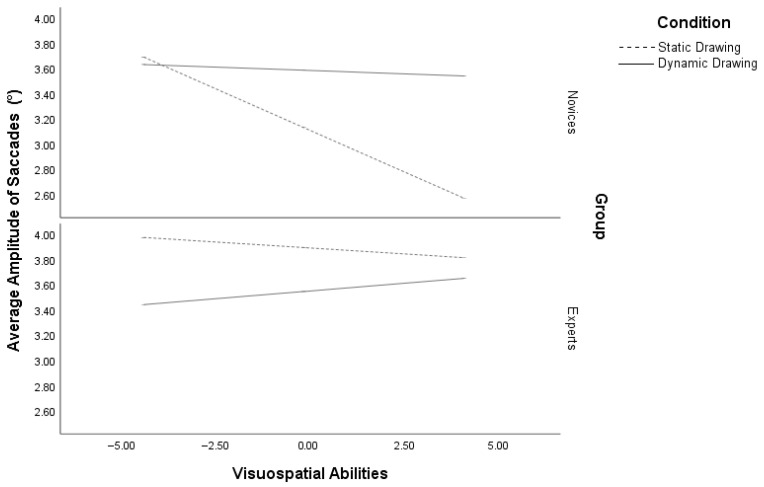
Moderation of average amplitude of saccades by visuospatial abilities and expertise in dynamic and static conditions.

The analysis of AAS indicated a significant regression model, R^2^ = 0.56, *p* < 0.001. The regression analysis demonstrated a main effect of condition [*β* = 1.29, se(HC4) = 0.25, *p* < 0.001], VSA [*β* = −0.44, se(HC4) = 0.09, *p* < 0.001] and expertise [*β* = 1.59, se(HC4) = 0.24, *p* < 0.001] on AAS. Participants in the dynamic drawing condition, in addition to those with low VSA and experts, had longer saccades compared to those in the static drawing condition with high VSA and novices, respectively. Furthermore, a significant three-way interaction was found between VSA, expertise, and condition [*β* = −0.07, se(HC4) = 0.03, *p* = 0.02]. While low VSA novices exhibited similar saccade amplitude across both conditions (*p* = 0.60), high VSA novices (*p* < 0.001) showed longer saccades in the dynamic drawing condition compared to the static condition. Experts with low VSA (*p* < 0.001) demonstrated longer saccades in the static drawings, whereas experts with high VSA displayed comparable saccade amplitude across both drawing conditions (*p* = 0.19) (Figure 5).

### 3.4. Average Peak Velocity of Saccades

**Figure 6:**
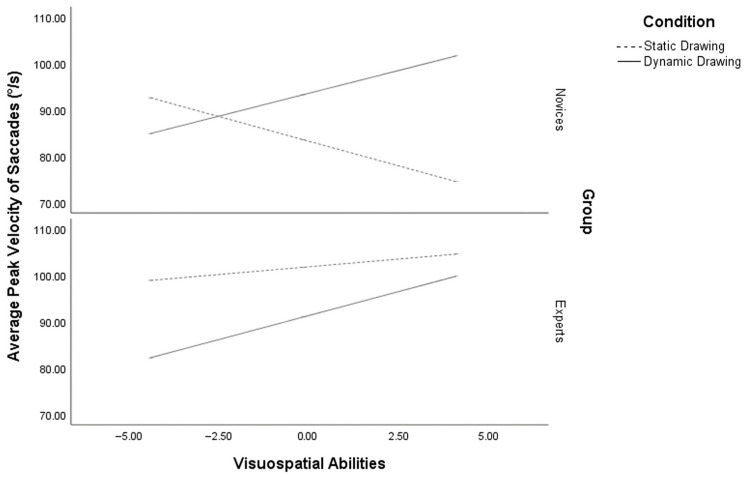
Moderation of average peak velocity of saccades by visuospatial abilities and expertise in dynamic and static conditions.

The analysis of APVS demonstrated a significant regression model, R^2^ = 0.50, *p* < 0.001. The regression analysis indicated a main effect of condition [*β* = 31.36, se(HC4) = 6.09, *p* < 0.001], VSA [ *β* = −11.67, se(HC4) = 2.39, *p* < 0.001] and expertise [*β* = 39.61, se(HC4) = 5.82, *p* < 0.001] on APVS. Participants exposed to the dynamic drawing condition, along with those with low VSA and experts, exhibited faster saccades than those in the static drawing condition with high VSA and novices, respectively. Notably, a significant three-way interaction was identified between VSA, expertise, and condition [*β* = −2.68, se(HC4) = 1.16, *p* = 0.02]. Novices with low VSA exhibited faster saccades in the static drawing condition (*p* < 0.01), whereas those with high VSA (*p* < 0.001) showed faster saccades in the dynamic drawing condition. Experts with low VSA (*p* < 0.001) demonstrated faster saccades in the static drawing condition, while experts with high VSA displayed comparable saccade velocity across both drawing conditions (*p* = 0.36) (Figure 6).

## 4. Discussion

This study explored the impact of drawing on the memorization and visual processing of a soccer tactical scene, taking into account individual differences in VSA and levels of expertise. The findings support our first hypothesis, showing no direct effect of the drawing condition on learning efficiency and suggesting that the instructional design may have increased the visual processing demands required during the task [14]. This finding may be attributed to the particular way multimedia principles were integrated into the design of the experimental conditions. Although dynamic drawing shares features with previous visual signaling interventions, the implementation of temporal contiguity and segmentation was somewhat distinctive. For instance, the temporal contiguity principle was reflected in the simultaneous verbal explanations that accompanied the gradual construction of diagrams, rather than in a sequential presentation of verbal and visual information [76]. Similarly, segmentation was determined by the coach’s pacing, rather than being controlled by the learner [77]. From another perspective, particularly that of observational learning, the inclusion of meaningful social cues, such as gestures, facial expressions, or gaze guidance, could further enhance the instructional value of dynamic drawing [16]. In the absence of these cues, the coach’s presence might offer limited support for deeper cognitive processing [5,78]. This was reflected in longer fixation durations and faster, longer saccades observed in the dynamic drawing condition. Learners rapidly redirected their gaze to follow the coach’s drawing gestures, indicating a higher level of attentional engagement [79,80,81]. However, the extended fixations on the diagram can be associated with higher cognitive processing difficulties [39,82,83,84,85,86].

In addition, regardless of the drawing condition, the study confirmed the second hypothesis, showing that individual differences influenced memorization and visual processing, with high VSA and expert participants outperforming their low VSA and novice counterparts. In accordance with the ability-as-enhancer hypothesis [30,31], participants with high VSA outperformed those with low VSA, suggesting that additional cognitive resources support more effective encoding and mental representation of visual information [27,35,51,78]. Eye-tracking data further supported this interpretation, revealing that VSA influenced visual processing strategies [87,88,89]. High VSA individuals exhibited longer fixations followed by shorter and slower saccades, indicating a focal processing mode associated with deeper engagement and better object retention [36,38,39]. In contrast, low VSA participants showed shorter fixations and longer, faster saccades, an ambient pattern typically linked to superficial scanning and less efficient integration of visual information [38,39,90]. Continuing within the framework of individual differences and focusing on the impact of expertise, experts demonstrated greater learning efficiency than novices in memorizing tactical scenes regardless of the drawing condition and confirmed previous findings [46,91]. This suggests that their extensive prior experience in soccer enabled them to better encode, store, and retrieve tactical information [27,51]. Eye movement analyses further supported this finding, revealing that experts showed longer fixations and longer, faster saccades, indicative of selective attention and a more strategic visual exploration [37,92,93].

More importantly, despite the absence of a direct effect of drawing conditions, considering individual characteristics such as VSA and expertise revealed a more nuanced understanding of the impact of drawing, confirming the third hypothesis regarding the interaction between drawing effectiveness and individual differences. Low VSA novices did not gain any compensatory advantage from the dynamic drawing condition, as their learning efficiency was comparable in both conditions. The evolving nature of dynamic drawing may have limited their capacity to extract, process, and integrate essential visual information. Eye-tracking data supported this finding, as low VSA participants exhibited longer fixation durations and slower saccades in the dynamic drawing condition, patterns typically associated with increased cognitive processing demands and difficulties in dealing with dynamically presented visual content [80,85,86,94]. Conversely, novices with high VSA benefited more from the dynamic drawing condition. As highlighted by Tlili et al. [14], this condition offers advantages for high VSA novices, who are better equipped to process and integrate dynamic visual information. They likely engaged more cognitive resources, enabling them to make better use of multimedia learning principles such as signaling, temporal contiguity, and segmentation [4,95,96]. In contrast, in the static condition, redundant information may have increased extraneous load and reduced their performance [97]. Eye-tracking data further support this, showing that high VSA participants processed diagrams more efficiently in the dynamic drawing condition, with shorter fixation durations and longer, faster saccades, suggesting rapid, targeted attention to relevant elements [94,98,99,100,101,102]. Even though the dynamic drawing condition enhanced learning efficiency for high VSA novices, the findings revealed an expertise reversal effect, where this dynamic drawing advantage shifted for experts and no longer offered the same benefit. Experts with low VSA benefited more from the static than the dynamic drawing condition. Indeed, instructional strategies designed to reduce extraneous cognitive load may become redundant for experts, leading to less efficient cognitive processing, greater working memory load, and ultimately hindering learning [46,103]. These findings can also be interpreted through the lens of skill acquisition theories, particularly the progression from controlled to automatic processing [104,105]. Experts, who operate largely within the autonomous stage of skill execution, rely on automatic and integrated knowledge structures that require minimal conscious effort. For these low VSA experts, the step-by-step and highly guided format of dynamic drawings may interfere with their fluid processing. In contrast, static drawings, presenting the entire scene at once, enable experts to engage with the material more globally and strategically without introducing unnecessary cognitive interference. This result is in line with previous research showing that circle signals added to diagrams no longer benefit expert learners as they can effectively direct their attention based on oral instructions alone, making such visual cues redundant [3]. This benefit of static drawing for low VSA experts is further supported by eye movement data, which show longer fixations as well as longer and faster saccades in this condition. This gaze pattern reflects selective attention allocation and an efficient visual span, as experts rapidly directed their gaze toward relevant information and fixated on it longer in order to construct an accurate mental representation [37,92,93]. On the other hand, while higher VSA may enhance the benefits of the drawing condition for novices, it reduced the drawing condition’s impact on learning efficiency for experts. High VSA experts appeared to benefit equally from both conditions and exhibited similar gaze patterns across them. Their ability to form clear mental representations in both dynamic and static formats suggests that, with increasing expertise, the influence of VSA on learning diminishes [33,106], as evidenced by equivalent visual processing across both conditions. This result aligns with Ben Mahfoudh and Zoudji [27], who demonstrated that, for experts, higher VSA reduce the performance gap between experimental conditions. It is also consistent with Fiorella et al. [13] who found that observing an instructor draw diagrams was particularly beneficial for students with low prior knowledge, whereas no significant advantage was observed for those with high prior knowledge.

While this study offers new insights into the role of coach-drawn diagrams in tactical memorization across VSA and expertise levels, several limitations should be acknowledged. Future research should explore how the instructor’s presence during the drawing process may either engage or distract learners. Investigating the use of social cues, such as gaze direction or pointing gestures, could help clarify their role in guiding attention and enhancing memorization. Moreover, as the study was conducted in a controlled laboratory setting, future studies should be carried out in more ecologically valid environments, such as real on-field soccer contexts, to increase the applicability of the findings. In addition, an important limitation related to our eye-tracking device and the reduced values of APVS observed in our data must be acknowledged. Although 50 Hz eye tracking has been used in several studies examining saccadic behavior [107], it nevertheless imposes clear constraints on the accurate estimation of saccadic kinematics. With a sampling interval of 20 ms, short saccades, particularly those in the 2–5° amplitude range observed in our present study, are represented by only one or two samples, which makes it difficult to capture the brief high-velocity phase of the saccade. This inherent temporal under-sampling necessarily leads to the attenuation of peak velocity observed in our study, even when saccadic amplitudes fall within the expected physiological range. This reduction may also be partly influenced by secondary effects of the Tobii I-VT filter which, although indispensable for reducing noise and stabilizing fixation and saccade segmentation, contributes to a smoothing of the velocity profile. The use of a 20 ms window for velocity estimation and a median noise-reduction procedure, both essential to minimize artefactual fluctuations, noise, and false saccades, and designed to preserve velocity peaks as much as possible, still inevitably reduces some high-frequency components of the signal and slightly reduces peak-velocity estimates. A further source of peak velocity attenuation arises specifically in the dynamic drawing condition, in which participants were exposed to continuous hand movements from the coach, likely eliciting smooth pursuit eye movements whose velocities can reach 30–100°/s. Although previous work has shown that sampling rates of 50–60 Hz can still capture clinically relevant saccadic parameters under dynamic conditions [108,109,110], the classifier may occasionally mislabel pursuit-related movements as slow saccades, since smooth pursuit velocities overlap with the artificially reduced saccadic peak velocities observed at 50 Hz, further contributing to the low velocity values. For these reasons, our APVS values should be interpreted as global tendencies rather than precise, highly robust measures of saccadic velocity. Future studies aiming to obtain accurate saccadic kinematics should employ eye trackers with higher sampling rates (≥250 Hz), which would minimize filter-related side effects, allow clearer differentiation between fixations, smooth pursuit, and saccades, and yield more robust and precise velocity estimates.

## 5. Conclusions

This study emphasizes the importance of considering individual differences, particularly VSA and expertise level, when using drawings to support tactical learning. From a practical standpoint, rather than presenting tactical diagrams based on personal preferences, coaches and educators are advised to first assess players’ VSA and determine their level of expertise. Based on these assessments, drawing-based instructional methods can then be adapted to each group. For novices with high VSA, coaches should draw the tactical diagram by hand step by step while providing verbal explanations. In this case, dynamic drawings are particularly beneficial, as they promote efficient visual search and deeper cognitive processing. In contrast, for other learners, and particularly for experts with low VSA, coaches are encouraged to verbally comment on static drawings, allowing them to integrate information at their own pace. By aligning instructional design with learners’ cognitive capacities and level of expertise, coaches can optimize visual attention, reduce cognitive overload, and improve the retention of tactical information.

## Figures and Tables

**Table 1:** Descriptive Statistics (Means and Standard Deviations) of the Dependent Variables by Drawing Conditions, Expertise groups, and VSA levels.

Condition	Group	VSA	LE	ADF	APVS	AAS
**Dynamic Drawing**	Experts	High (14–18)	1.2 (1)	379.6 (75.1)	95.9 (6.7)	3.6 (0.2)
		Low (5.5–9.25)	−0.3 (1.2)	291.2 (38.8)	80.6 (8.7)	3.5 (0.2)
	Novices	High (14–18)	1.1 (0.7)	325.8 (93.7)	102.5 (14.1)	3.5 (0.2)
		Low (3.5–9)	−1.6 (1.2)	488.8 (140.9)	85.3 (10.7)	3.6 (0.2)
**Static Drawing**	Experts	High (14.25–18.5)	1.4 (0.9)	395.8 (28.7)	108.2 (17.2)	3.8 (0.2)
		Low (5.5–9)	1.3 (0.3)	421.5 (76.1)	107.1 (3.8)	4.1 (0.1)
	Novices	High (14–19)	−1.9 (0.8)	442.5 (38.9)	73.1 (4.7)	2.5 (0.3)
		Low (4–8.5)	−0.6 (1)	226.4 (58.9)	96.7 (4.1)	3.8 (0.3)

Note. LE = Learning Efficiency; ADF = Average Duration of Fixation; APVS = Average Peak Velocity of Saccades; AAS = Average Amplitude of Saccades.

## Data Availability

All data and analyses can be downloaded from the Open Science Framework: https://osf.io/qkhp6/overview?view_only=adbf6e1e70f84954918ddc65c2720e16 (accessed on 15 October 2025).
